# Early Assessment of the Efficacy of Temozolomide Chemotherapy in Experimental Glioblastoma Using [^18^F]FLT-PET Imaging

**DOI:** 10.1371/journal.pone.0067911

**Published:** 2013-07-04

**Authors:** Thomas Viel, Sonja Schelhaas, Stefan Wagner, Lydia Wachsmuth, Katrin Schwegmann, Michael Kuhlmann, Cornelius Faber, Klaus Kopka, Michael Schäfers, Andreas H. Jacobs

**Affiliations:** 1 European Institute for Molecular Imaging (EIMI), Westfälische Wilhelms-University (WWU), Münster, Germany; 2 Department of Nuclear Medicine, University Hospital Münster, Westfälische Wilhelms-University (WWU), Münster, Germany; 3 Department of Radiology, University Hospital Münster, Westfälische Wilhelms-University (WWU), Münster, Germany; 4 Interdisciplinary Centre of Clinical Research (IZKF), Westfälische Wilhelms-University (WWU), Münster, Germany; 5 Radiopharmaceutical Chemistry, German Cancer Research Center (dkfz), Heidelberg, Germany; 6 Department of Geriatric Medicine, Evangelische Kliniken, Johanniter Krankenhaus, Bonn, Germany; Wayne State University, United States of America

## Abstract

Addition of temozolomide (TMZ) to radiation therapy is the standard treatment for patients with glioblastoma (GBM). However, there is uncertainty regarding the effectiveness of TMZ. Considering the rapid evolution of the disease, methods to assess TMZ efficacy early during treatment would be of great benefit. Our aim was to monitor early effects of TMZ in a mouse model of GBM using positron emission tomography (PET) with 3′-deoxy-3′-[^18^F]fluorothymidine ([^18^F]FLT).

**Methods:**

Human glioma cells sensitive to TMZ (Gli36dEGFR-1) were treated with sub-lethal doses of TMZ to obtain cells with lower sensitivity to TMZ (Gli36dEGFR-2), as measured by growth and clonogenic assays. Gli36dEGFR-1 and Gli36dEGFR-2 cells were subcutaneously (s.c.) or intracranially (i.c.) xenografted into nude mice. Mice were treated for 7 days with daily injection of 25 or 50 mg/kg TMZ. Treatment efficacy was measured using [^18^F]FLT-PET before treatment and after 2 days. Computed Tomography (CT) or Magnetic Resonance Imaging (MRI) were used to determine tumor volumes before treatment and after 7 days.

**Results:**

A significant difference was observed between TMZ and DMSO treated tumors in terms of variations of [^18^F]FLT T/B ratio as soon as day 2 in the i.c. as well as in the s.c. mouse model. Variations of [^18^F]FLT T/B uptake ratio between days 0 and 2 correlated with variations of tumor size between days 0 and 7 (s.c. model: n_tumor_ = 17 in n_mice_ = 11, *P*<0.01; i.c. model: n_tumor/mice_ = 9, *P*<0.01).

**Conclusions:**

Our results indicate that [^18^F]FLT-PET may be useful for an early evaluation of the response of GBM to TMZ chemotherapy in patients with glioma.

## Introduction

Primary malignant central nervous system tumors represent about 2% of all cancers and account for a high rate of morbidity and mortality. They are the leading cause of death from solid tumors in children and the third leading cause of death from cancer in adolescents and adults aged 15 to 34 years [1]. Among malignant gliomas, glioblastomas (GBM) are the most common and fatal neoplasms, representing approximately 50% of all gliomas and 24% of all primary intracranial tumors [2]. Resection followed by combined radiotherapy and temozolomide (TMZ) chemotherapy is the standard therapy protocol for patients suffering from GBM [3]–[5]. However, there is considerable uncertainty with regards to the effectiveness of TMZ. Overexpression of the DNA repair enzyme O6-methylguanine-DNA methyltransferase (MGMT), which antagonizes the genotoxic effects of alkylating agents like TMZ, is recognized as an unfavorable prognostic marker of the efficacy of alkylating chemotherapy [6], [7]. Nevertheless, factors other than MGMT expression may also be involved in TMZ resistance, such as over-expression of the mismatch repair system or attenuation of wild-type p53 function [8].

Assessment of treatment efficacy is usually performed employing gadolinium-enhanced magnetic resonance imaging (MRI) in 2–3 months intervals during treatment [4]. However, conventional imaging techniques usually fail to detect effects of radio- and chemotherapy at early time points because morphologic treatment effects may be visible only after several weeks or months [9]–[11]. Early assessment of tumor response to therapy offers the opportunity to tailor or intensify therapy in patients where first therapeutic approaches failed. Positron emission tomography (PET) allows the assessment of changes at the molecular level and may offer the possibility of detection of tumor response to therapy at a relatively early stage [9], [12]–[14]. The PET tracer 3′-deoxy-3′-[^18^F]fluorothymidine ([^18^F]FLT) was introduced in 1998 by Shields et al. in order to non-invasively visualize cell proliferation [15]. [^18^F]FLT is an analog to thymidine retained in the cell after phosphorylation by thymidine kinase 1 (TK1). [^18^F]FLT accumulation depends on the DNA synthesis rate and has been shown to correlate with TK1 expression and with the percentage of cells in S phase of the cell cycle [16]. [^18^F]FLT has been validated in several clinical studies to assess proliferation of different types of tumors [17]–[19]. Due to low uptake in normal brain, [^18^F]FLT is a promising tracer to evaluate tumor grade and cellular proliferation in brain tumors [12], [20]–[21], and could be of particular interest for the read-out of TMZ therapy.

In this study, our goal was to investigate whether early effects of TMZ chemotherapy on GBM proliferation could be detected using [^18^F]FLT-PET imaging. We measured the variation of [^18^F]FLT tumor-to-background (T/B) uptake ratio after 2 days of TMZ treatment and observed a significant positive correlation with the variation of size observed at a later time point (day 7).

## Methods

### Cell Culture

Human Gli36dEGFR glioma cells (kind gift of Dr. David Louis, Molecular Neurooncology Laboratory, MGH, Boston, MA) [22]–[23] were established by retroviral transfer of a mutant epidermal growth factor receptor (Δ_2–7_EGFR) into the human Gli36 glioma cell line, enhancing its tumorigenic capacity. Cells were grown as monolayer in Dulbecco’s Modified Eagle Medium (DMEM) with high glucose and GlutaMAX (Gibco) supplemented with 10% Fetal Bovine Serum (FBS; Invitrogen), and 1% Penicillin/Streptomycin (P/S; PAA Laboratories) at 37°C in a 5% CO_2_/95% air atmosphere.

Gli36dEGFR cells were called Gli36dEGFR-1 in order to distinguish them from the cell line obtained after treatment with TMZ (Sigma-Aldrich). Gli36dEGFR-1 cells were cultivated in presence of 50 µM TMZ for one month. During the first days of the treatment extensive cell death was observed. Surviving cell clones were amplified. After one month, TMZ treated cells presented a growth rate similar to DMSO treated cells. They were further cultivated in normal media and termed Gli36dEGFR-2.

### Cell Culture Assays

Acute growth inhibition/cytotoxicity assays involved the exposure of the Gli36dEGFR-1 and Gli36dEGFR-2 cells seeded at 2.5×10^4^ cells/well in 24-well plates to increasing concentrations (0–2000 µM) of TMZ for 72 h. Cells were counted using a Z2 coulter particle count and size analyzer (Beckman Coulter).

Clonogenic survival assays were performed by seeding 1000 cells/well in six-well plates and exposing them to increasing concentrations of TMZ (0–2000 µM), followed by further observation for 7 days. The number of colonies was assessed using crystal violet (Roth) staining.

Caspase activation assays were performed using the caspase substrate Ac-DEVD-AMC (Axxora). After treatment of cells with 75 µM TMZ and incubation for 0, 6, 12, 24, 48 and 72 h, total proteins were extracted using a commercial lysis buffer (Cell Signaling) complemented with phosphatase inhibitors (Phosphatase Inhibitor Cocktail; Calbiochem), and the amount of protein present in each sample was quantified using a Bio-Rad protein assay (Bio-Rad Laboratories). Protein extracts were incubated for 120 min at 37°C with the caspase substrate Ac-DEVD-AMC. Caspase activation was assessed by measuring the accumulation of the cleaved fluorogenic product AMC with a Fusion universal microplate analyzer (Perkin Elmer) at excitation and emission wavelengths of 360 nm and 460 nm, respectively.

### Western Blot Analysis

The expression of MGMT and MSH6 proteins was evaluated using western blotting. Cells were lysed and protein concentrations were determined as for the caspase activation assay. Equal amounts of denatured (95°C; 5 min) protein were loaded onto a 12% (MGMT) or 6% (MSH6) Tris-glycin sodium dodecyl-sulfate polyacrylamide gel. After separation (2 h; 140 V), proteins were blotted to a nitrocellulose membrane (Protran, Whatman) using a semi-dry blotting system (1 h, 120 mA; Biometra). After blocking nonspecific binding with 3% Bovine Serum Albumine Fraction V (BSA; PPA Laboratories) dissolved in Tris-buffered saline containing 0.1% Tween 20 (TBST) for 1 h, the membrane was washed with TBST and probed with a specific primary antibody. After washing, the membrane was incubated with horseradish peroxidase conjugated secondary antibody in TBST for 1 h. After three washing steps (TBST, 15 min), protein detection was achieved through chemiluminescent reaction using the Pierce ECL Plus Western Blotting Substrate (Pierce Biotechnology).

The following antibodies were used: primary monoclonal mouse anti-MGMT (1∶250 dilution in 3% TBST-BSA solution; MT3.1, ab39253, Abcam), primary monoclonal rabbit anti-MSH6 (1∶500 dilution in 3% TBST-BSA solution; ab92471, Abcam), primary monoclonal mouse anti-actin clone C4 (1∶1000 dilution in 3% TBST-BSA solution; 69100, MP Biomedicals), secondary polyclonal rabbit anti-mouse immunoglobulins (1∶5000 dilution in TBST solution; P0260, Dako), secondary polyclonal goat anti-rabbit immunoglobulins (1∶2000 dilution in TBST solution; P0448, Dako).

### Animal Experiments

All experiments performed in the study were in accordance with the German Law on the Care and Use of Laboratory Animals and approved by the Landesamt für Natur, Umwelt und Verbraucherschutz Nordrhein-Westfalen (LANUV). Female NMRI nude mice were housed at constant temperature (23°C) and relative humidity (40%), under a regular light/dark schedule. Food and water were freely available. Nude mice (n = 35) were injected subcutaneously (s.c.) with 1×10^6^ Gli36dEGFR-1 and Gli36dEGFR-2 cells suspended in 50 µl of DMEM without serum and antibiotics (plain DMEM). Two Gli36dEGFR-1 (left shoulder and upper back) and two Gli36dEGFR-2 (right shoulder and lower back) xenografts were implanted in each nude mouse. Usually two or three xenografts were observed per mouse. Gli36dEGFR-1 cells were also stereotactically implanted into the right striatum of nude mice (n = 10). Anesthetized animals were placed in a stereotactic head frame. A midline incision of the skin was made, and a small hole was drilled in the skull at the appropriate location. 5×10^5^ cells in 2 µl of plain DMEM were intracranially (i.c.) injected with a 10 µl Hamilton syringe at the following coordinates: lateral - 2.0 mm, dorsoventral −3.0 mm, using the bregma as a reference. After the injection, the needle was left in place for an additional 5 minutes before being slowly withdrawn from the brain.

### Treatment and Imaging Protocol

12 days after tumor implantation nude mice were treated with daily injection of DMSO, 25 mg/kg TMZ or 50 mg/kg TMZ in DMSO in the intra-peritoneal cavity (40 µl) for 7 days.

Tumor response to TMZ treatment was measured using small animal [^18^F]FLT-PET/CT imaging before starting the treatment as well as after 2 and 7 days of treatment. Sub-cutaneous tumor sizes were measured using the CT scan performed after each PET scan. For intracranial tumors, sizes were measured using T2-weighted (T2w) MR images. T2w MR scans were performed one day before treatment (day −1) as well as on day 6. At day 2 or 7 mice were sacrificed for histological analyses, and tumors were placed in 4% paraformaldehyde (PFA). Nude mice bearing intracranial tumors were first perfused using 4% PFA.

### Radiosynthesis of [^18^F]FLT

No-carrier-added aqueous [^18^F]fluoride ions were produced on a RDS 111e cyclotron (CTI-Siemens) by irradiation of 97% enriched [^18^O]water using 10 MeV proton beams. Radiosynthesis of [^18^F]FLT was carried out performed as described previously [24] using an automated PET tracer synthesizer (TRACERLab Fx FN Synthesizer; GE Healthcare). Separation and purification of the radiosynthesized compounds were performed within the radiosynthesizer by radio-RP-HPLC using a Knauer K-1800 pump (flow = 7 ml/min, eluent: water for injection/EtOH 97/3 (v/v), a Knauer K-2501 UV-detector (λ = 254 nm), and a Nucleosil 100–10 C18 column (250 x 8 mm^2^). The product fraction of [^18^F]FLT (retention time t_R = _6.0±0.6 min) was collected. Finally, the [^18^F]FLT solution was dispensed into a sterile pyrogen-free 20 ml glass vial by passing through a 0.22 µm sterile filter (Millipore Millex FG). The total activity of [^18^F]FLT at the end of the radiosynthesis was 1.2–5.6 GBq (2.9±1.8 GBq).

### Small Animal PET/CT

Mice were anaesthetized with 2% isoflurane (DeltaSelect; Dreieich, Germany) in O_2_, and a lateral tail vein catheter was positioned using a 27 G needle connected to a 15 cm polyethylene catheter tubing. 12 MBq 3′-deoxy-3′-[^18^F]fluorothymidine ([^18^F]FLT) were injected as a bolus (100 µl of [^18^F]FLT solution flushed with 100 µl saline) via the tail vein, and subsequent PET scanning was performed. PET experiments were carried out using a high resolution small animal scanner (32 module quadHIDAC, spatial resolution of 0.7 mm using iterative EM reconstruction including resolution recovery; Oxford Positron Systems Ltd., Oxford, UK) with uniform spatial resolution (<1 mm) over a large cylindrical field (165 mm diameter, 280 mm axial length) [25]. List-mode data were acquired for 20 min starting 70 min after tracer injection. Subsequently, the scanning bed was transferred to the Computed Tomography (CT) scanner (Inveon, Siemens Medical Solutions, USA) and a CT acquisition with a spatial resolution of 80 µm was performed for each mouse.

### Small Animal MRI

Mice were anaesthetized with 2% isoflurane in O_2_. MRI was performed with a 9.4 T small animal MR scanner with 20 cm bore size (Bio-Spec 94/20; Bruker BioSpin MRI GmbH, Germany). The system was operated using the software ParaVision 5.1. (Bruker BioSpin MRI GmbH). We used the helium-cooled Cryoprobe (Bruker BioSpin MRI GmbH) to obtain anatomical 2D T2w RARE brain images in the coronal plane (TR/TE 4000/51 ms, RARE factor 8, FOV 2 cm, 256^2^ matrix, 0.8 mm slice thickness, 20 slices, fat suppression, scan time 8 min).

### Data Analysis

PET data were reconstructed into a static frame using an iterative reconstruction algorithm. Reconstructed PET and CT image data sets were co-registered based on extrinsic markers attached to the multimodal scanning bed (image analysis software Inveon Research Workplace 3.0; Siemens Medical Solutions, USA). PET/CT and MR images were co-registered using contours of the skull and head of the mice using the software VINCI (http://www.nf.mpg.de/vinci3/) [26]. MR and CT images were used to delineate the contour of the tumors and to measure their volumes. A volume-of-interest (VOI) approach was used to determine the maximal radiotracer uptake in the tumor ([^18^F]FLTmax). To calculate tumor-to-background uptake ratios ([^18^F]FLT T/B), the tumor [^18^F]FLTmax was divided by the mean [^18^F]FLT uptake in reference regions. The reference region was the muscle of the lower left leg for the s.c. xenografts and the mirror region of the tumors drawn in the contralateral hemisphere for the i.c. xenografts.

### Staining of Paraffin Sections

Depending on the experimental setup, mice were sacrificed after 2 or 7 days of TMZ treatment. The tumors were excised, fixed in 4% PFA, embedded in paraffin, cut in 5 µm sections and prepared for immuno-histological analysis. After rehydrating and heat-induced epitope retrieval for 30 min in citrate buffer (pH 6.0), sections were incubated in peroxidase blocking solution (S3022; DAKO, Germany) for 5 min and treated with serum-blocking solution for 15 min. Then, sections were incubated overnight at 4°C with the rabbit monoclonal primary antibodies (Ki67: dilution 1∶100, ab16667, Abcam; TK1: dilution 1∶200, EPR3193, ab76495, Abcam; active caspase-3: dilution 1∶100, Clone C92–605, nr. 559565, BD Pharmingen Inc.). Labeling of the primary antibody was performed using a commercial avidin-biotin complex detection kit based on a biotinylated anti-rabbit antibody (Ki67 and TK1: goat anti-rabbit dilution 1∶500, B21078, Invitrogen; active caspase-3: donkey anti-rabbit dilution 1∶200, RPN1004, Amersham Inc.) according to the manufacturers manual, followed by incubation with 3,3′-diaminobenzidine (DAB, D-5637; Sigma) for 5 min. Sections were counterstained with hematoxylin, dehydrated and mounted using Entellan (Merck, Germany). Histological analysis was performed using a Nikon Eclipse 90i light microscope (Nikon). Quantification of Ki67, TK1 and active caspase-3 staining (3 tumors per group, 3 different areas per tumor) was performed employing the Fiji software (http://fiji.sc/Fiji). Color images were deconvolved (H-DAB color deconvolution). The resulting brown mono-chrome images were thresholded and the resulting images were submitted to the “Watershed” plugin. Thereafter, the number of positive particles per field of view (for Ki67 and active caspase-3) or the percent of area occupied by the positive pixels (for TK1) were quantified using the “Analyze particles” plugin. Mean values were normalized to the mean values of the DMSO treated Gli36dEGFR-1 tumors.

### Statistical Analysis

Means±standard deviation (SD) of T/B ratios of radiotracer uptake were calculated. Student’s T-Test, Mann-Whitney Rank Sum Test, One Way Anova, and Kruskal-Wallis One Way Analysis were performed using SigmaStat 3.0 (SPSS, Inc., Chicago, IL) to assess significant alterations over time between two groups. Correlation coefficients and significance were determined using the Pearson correlation analysis using the same software.

## Results

### Imaging Response of s.c. glioma Xenografts to TMZ Using Small Animal [^18^F]FLT-PET/CT

**Figure 1:**
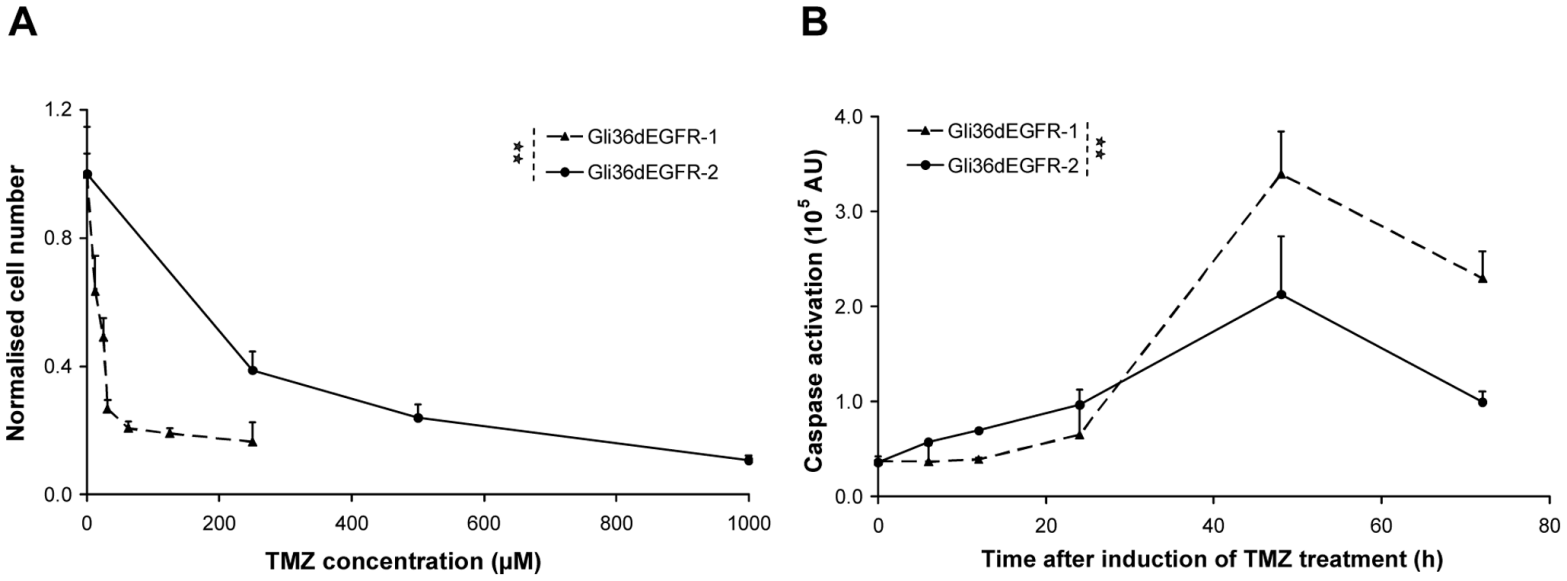
Temozolomide mediated cytotoxicity in human Gli36dEGFR-1 and Gli36dEGFR-2 glioma cells. **A.** Growth inhibition assay showed that Gli36dEGFR-2 cells were less sensitive towards TMZ than Gli36dEGFR-1 (*P*<0.001, Two-Way ANOVA). **B.** Both cell lines showed caspase activation following single treatment with 75 µM TMZ. This activation was less pronounced for Gli36dEGFR-2 (difference significant; **: *P*<0.001, Two-Way ANOVA).

The goal of the study was to evaluate [^18^F]FLT-PET as an early marker of the response of GBM xenografts to TMZ treatment. In order to have some diversity in the tumor response, we first treated human Gli36dEGFR glioma cells (named Gli36dEGFR-1), presenting a high sensitivity to TMZ, with sub-lethal doses of TMZ (50 µM) to obtain Gli36dEGFR-2 cells. Treatment of both cells with a single dose of TMZ resulted in caspase activation after 48 h. However, caspase activation was found to be lower in the Gli36dEGFR-2 cells compared to Gli36dEGFR-1 cells (**Figure 1** **)**. Growth and clonogenic assays (**Figure 1** **and Figure S1**) confirmed the lower sensitivity to TMZ of the Gli36dEGFR-2 cells as compared to Gli36dEGFR-1 cells. Gli36dEGFR-1 and Gli36dEGFR-2 glioma cells were s.c. xenografted into nude mice. Small animal [^18^F]FLT-PET/CT was performed before and after 7 days of treatment with either DMSO, 25 mg/kg TMZ or 50 mg/kg TMZ (**Figure S2**). Variation of size between days 0 and 7 showed that both Gli36dEGFR-1 and Gli36dEGFR-2 xenografts responded to TMZ treatment in a dose dependent manner (Kruskal-Wallis One Way Analysis; *P* = 0.002, *P* = 0.006, respectively). At day 7, FLT tumor accumulation was affected in treated xenografts in a dose dependent manner, but the difference between the different treatment regimens was significant only for the Gli36dEGFR-1 and not for the Gli36dEGFR-2 xenografts (Kruskal-Wallis One Way Analysis; *P* = 0.006, *P* = 0.114, respectively).

**Figure 2:**
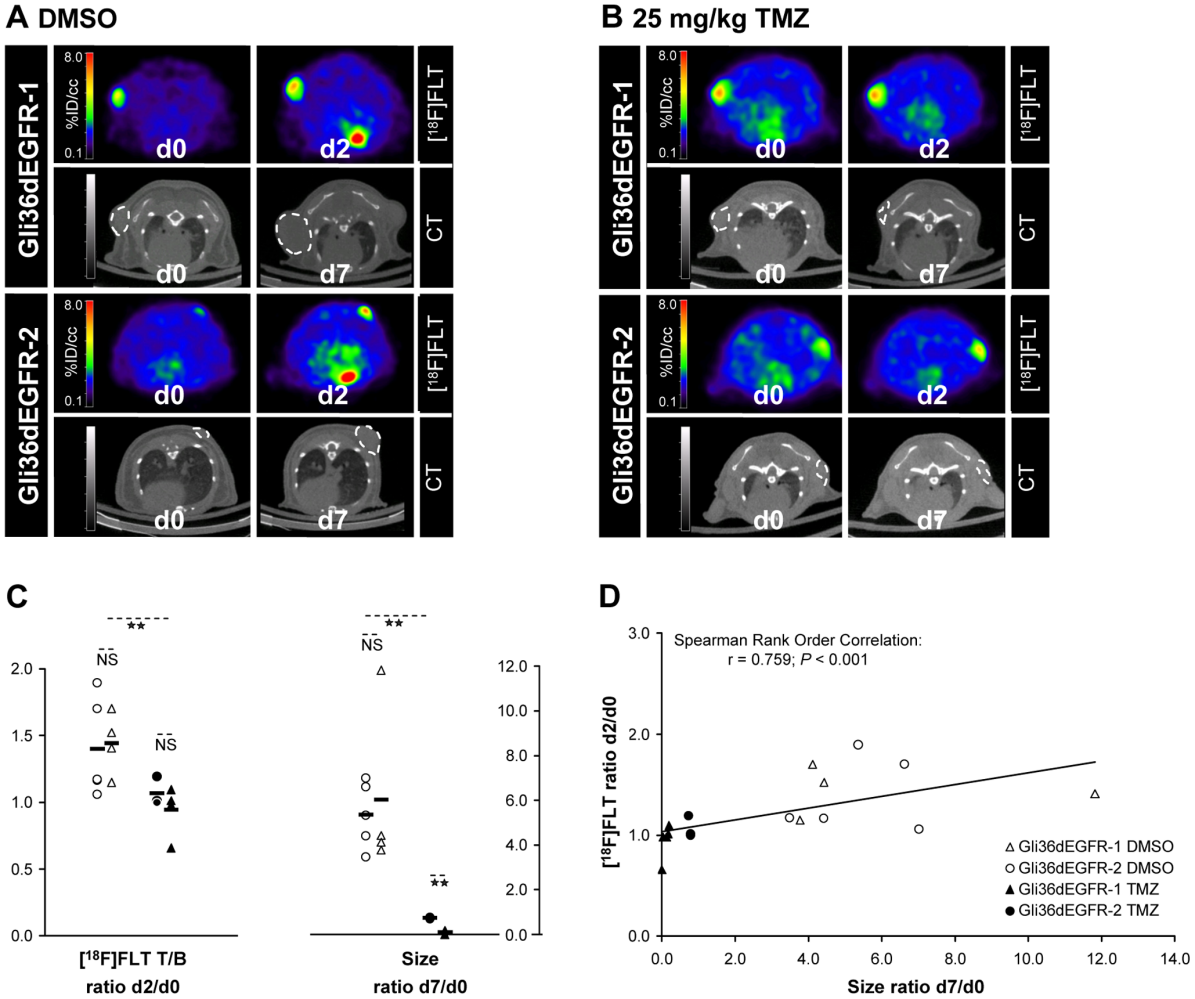
[^18^F]FLT-PET indicates GBM response to TMZ after 2 days of treatment in s.c. xenografts. [^18^F]FLT-PET scans were acquired in mice bearing Gli36dEGFR-1 and Gli36dEGFR-2 xenografts before and after 2 days of TMZ treatment. CT scans were acquired at day 0 and day 7 in order to determine the tumor size. [^18^F]FLT-PET at days 0 and 2 and corresponding CT images at days 0 and 7 of representative mice bearing Gli36dEGFR-1 and Gli36dEGFR-2 xenografts and receiving daily injection of DMSO (**A**) or of 25 mg/kg TMZ (**B**) are presented. **C.** Quantitative analysis of changes of [^18^F]FLT T/B ratios between days 0 and 2 and changes of CT-determined size between days 0 and 7 in mice receiving daily injection of DMSO (Gli36dEGFR-1 n_tumor_ = 4 in n_mice_ = 4; Gli36dEGFR-2 n_tumor_ = 5 in n_mice_ = 5) or of 25 mg/kg TMZ (Gli36dEGFR-1 n_tumor_ = 5 in n_mice_ = 4; Gli36dEGFR-2 n_tumor_ = 3 in n_mice_ = 3). Differences between the treated and control groups were tested for significance using T-Test or Mann-Whitney Rank Sum Test (**: *P*<0.01, NS: not significant). Increased [^18^F]FLT tracer accumulation was observed in DMSO treated Gli36dEGFR-1 and Gli36dEGFR-2 xenografts between day 0 and day 2. [^18^F]FLT accumulation was constant (or slightly reduced) in treated Gli36dEGFR-1 xenografts. [^18^F]FLT accumulation in treated Gli36dEGFR-2 xenografts were only slightly increased at day 2 compared to day 0. **D.** A significant positive correlation (Spearman correlation analysis; r = 0.759; *P*<0.001) was observed between changes in [^18^F]FLT T/B uptake ratio between day 0 and day 2 and changes in size between day 0 and day 7.

To be closer to the clinical situation, and in order to determine whether [^18^F]FLT-PET imaging is able to predict glioma response to TMZ chemotherapy as early as at the second day of treatment, small animal [^18^F]FLT-PET/CT scans were performed before and after 2 days of treatment with DMSO or 25 mg/kg TMZ (**Figure 2**). Additional CT scans were obtained after one week of daily treatment in order to determine the tumor size at day 7 and the correlation between the variations of [^18^F]FLT T/B ratio between days 0 and 2 and the variations of size between days 0 and 7 was investigated. A significant difference (Mann-Whitney Rank Sum Test, *P* = 0.003) was observed using [^18^F]FLT-PET between DMSO and TMZ treated tumors after 2 days of treatment. [^18^F]FLT T/B ratios were increased between day 0 and day 2 for the DMSO treated tumors (ratio d2/d0∶1.42±0.30), while remaining constant for the treated tumors (ratio d2/d0∶0.99±0.15). Furthermore, a positive correlation was observed between variation of size between days 0 and 7 and variation of [^18^F]FLT T/B ratio between days 0 and 2 (linear correlation; Spearman Rank Order Correlation, r = 0.759, *P*<0.0001). At this early time point, the difference between TMZ treated and DMSO treated tumors in terms of CT-determined size variation was not significant and the correlation between the size ratio d2/d0 and the size ratio d7/d0 did not reach the significant level either (**Figure S3**). However, despite the fact that the mean tumor size variation observed between days 0 and 7 was significantly different for the treated Gli36dEGFR-1 and the treated Gli36dEGFR-2 xenografts (ratio d7/d0∶0.10±0.09 for the Gli36dEGFR-1 vs. 0.76±0.03 for the Gli36dEGFR-2 xenografts; *P*<0.001, T-Test), the mean variation of [^18^F]FLT T/B ratio between days 0 and 2 was not significantly different between the two treated tumor groups (ratio d2/d0∶0.94±0.17 for the Gli36dEGFR-1 vs. 1.07±0.11 for the Gli36dEGFR-2 xenografts; non-significant).

**Figure 3:**
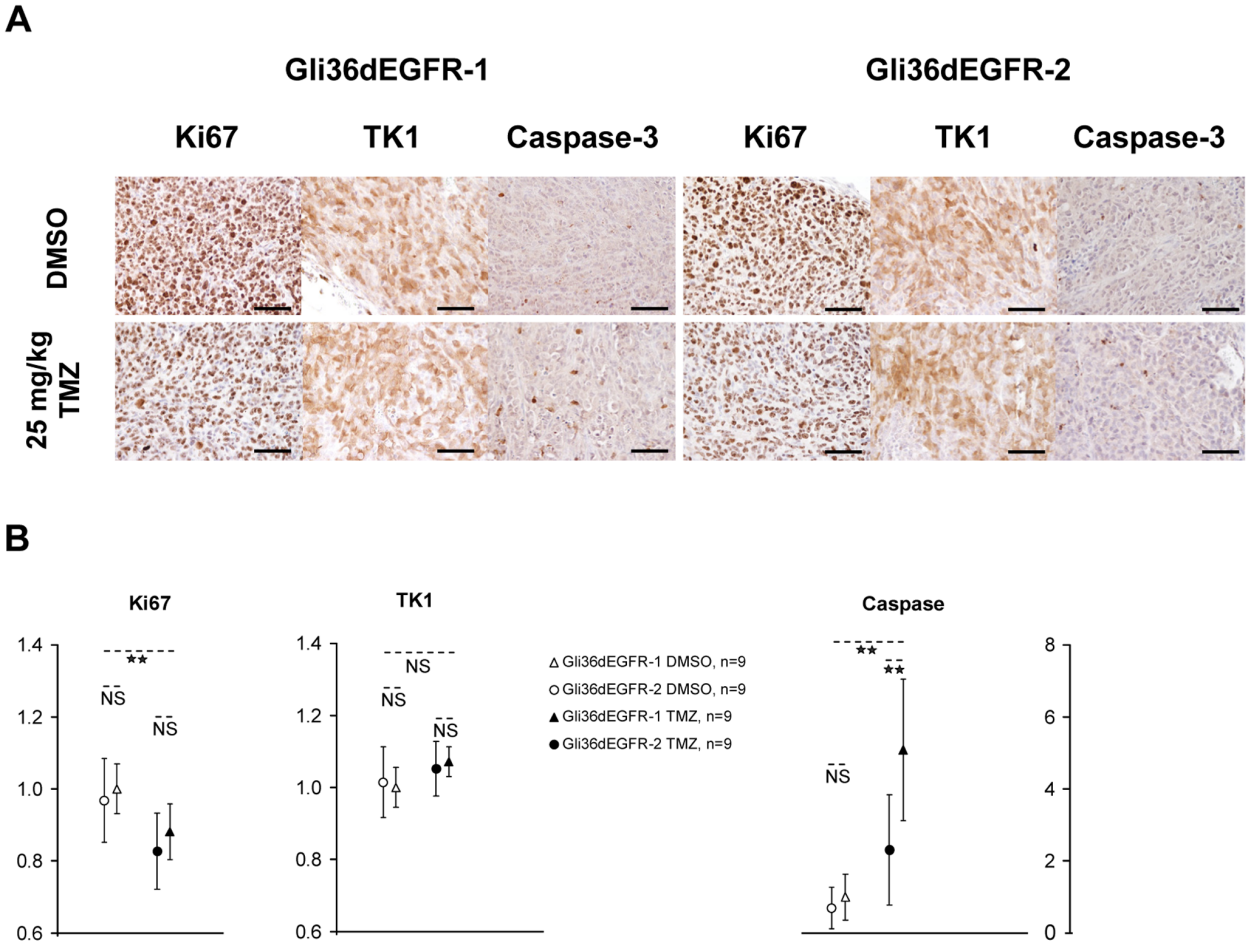
Immunohistochemistry of Gli36dEGFR-1 and Gli36dEGFR-2 tumor tissue for Ki67, TK1 and active caspase-3 expression after 2 days of treatment. **A.** Tumor tissue sections were stained for Ki67, TK1 and active caspase-3 after 2 days of DMSO or 25 mg/kg TMZ treatment. Scale bars = 50 µm. **B.** Quantification of Ki67, TK1 and Active caspase-3 staining. Differences between the treated (black; Gli36dEGFR-1: triangle, Gli36dEGFR-2: circle) and the control (white; Gli36dEGFR-1: triangle, Gli36dEGFR-2: circle) groups were tested for significance using T-Test or Mann-Whitney Rank Sum Test (**: *P*<0.01, NS: not significant). Ki67 positive cells were less numerous in tumors treated with TMZ vs. DMSO. No difference could be observed on the TK1 staining. Active caspase-3 was induced in TMZ treated tumors, especially in Gli36dEGFR-1 treated xenografts.

Immunostaining for Ki67 at day 2 showed a significant reduction of the Ki67 positive cells in TMZ vs. DMSO treated tumors, in good agreement with the [^18^F]FLT-PET images (**Figure 3**). No significant difference could be observed between Gli36dEGFR-1 and -2 at this early stage. No change in TK1 staining could be observed. Active caspase-3 was significantly up-regulated in TMZ vs. DMSO treated tumors, and in treated Gli36dEGFR-1 vs. treated Gli36dEGFR-2 tumors.

### Early Response of i.c. glioma Xenografts to TMZ as Assessed by Small Animal [^18^F]FLT-PET/MR Imaging

**Figure 4:**
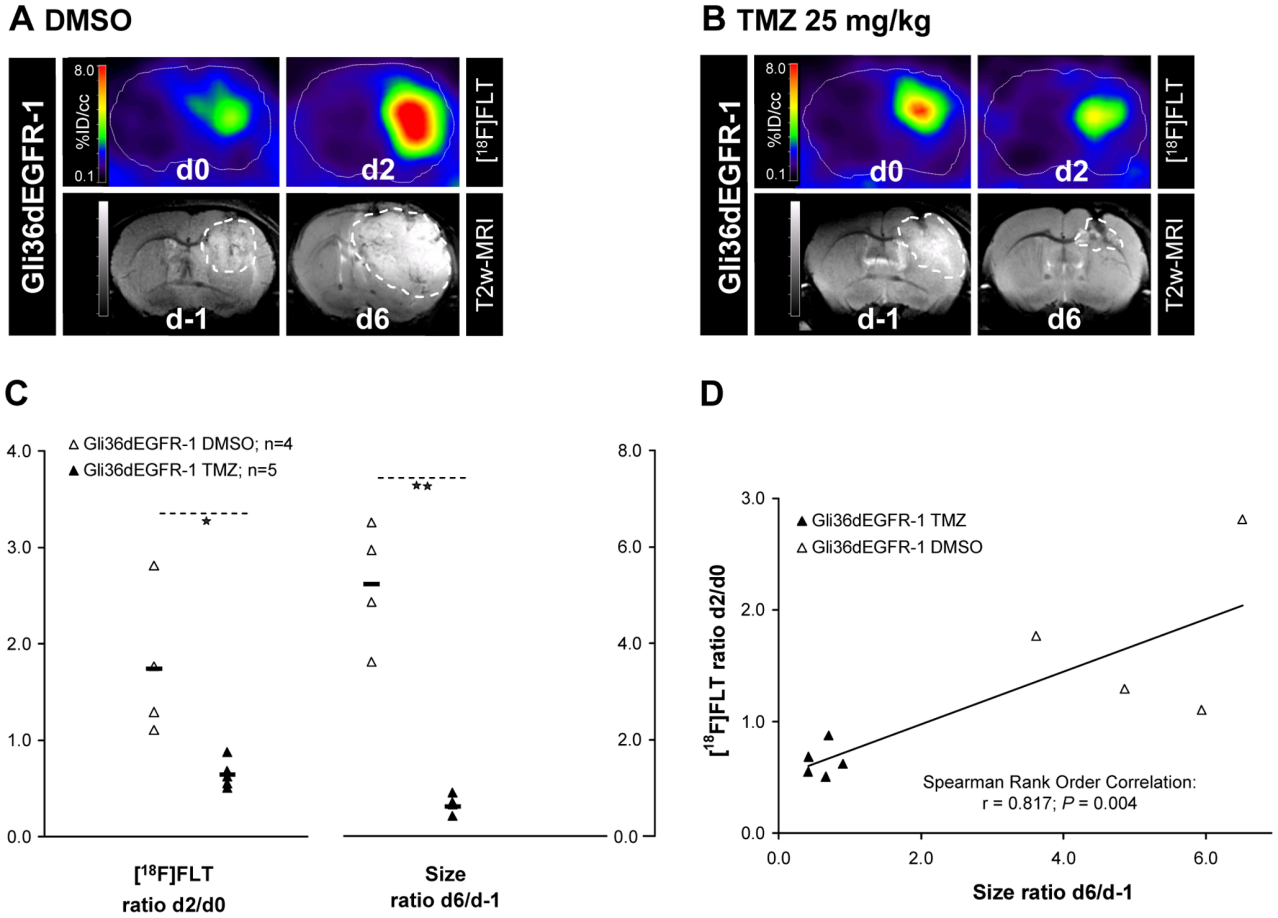
[^18^F]FLT-PET indicates response of i.c. growing gliomas to TMZ after 2 days of treatment. Representative [^18^F]FLT-PET images at days 0 and 2 and corresponding T2w-MR images at day -1 and day 6 of two mice bearing an orthotopic i.c. Gli36dEGFR-1 xenograft and receiving daily injection of DMSO (**A)** or 25 mg/kg TMZ (**B**). [^18^F]FLT accumulation was reduced in Gli36dEGFR-1 xenografts in response to treatment with TMZ. **C.** Quantitative analysis of changes in [^18^F]FLT T/B uptake ratio after 2 days (as determined by PET) and in size after 6 days (as determined by T2w-MRI) of daily injection of DMSO (n_tumor_ = 4) and 25 mg/kg TMZ (n_tumor_ = 5) for the Gli36dEGFR-1 i.c. xenografts. Differences between the treated and control groups were tested for significance using T-Test or Mann-Whitney Rank Sum Test (*: *P*<0.05; **: *P*<0.01). **D.** A positive correlation (Spearman rank correlation analysis) was observed between changes in [^18^F]FLT T/B uptake ratio between day 0 and day 2 and changes in size between day -1 and day 6.

To analyze treatment effects in intracranial growing gliomas, Gli36dEGFR-1 cells were implanted i.c. into the striatum of nude mice (n = 10) and small animal [^18^F]FLT-PET/CT scans were performed before and after 2 days of treatment with DMSO or 25 mg/kg TMZ (**Figure 4**). T2w MR scans were performed one day before beginning of treatment (day -1) as well as at day 6 in order to estimate the variation in tumor size. In mice receiving DMSO (n = 4), [^18^F]FLT T/B ratio increased between day 0 and day 2 (increase of 70±80%). In contrast, after treatment with 25 mg/kg TMZ (n = 5) the [^18^F]FLT T/B ratio was significantly reduced by 40±10% (*P* = 0.015, T-Test). A positive correlation was observed between the change in size from day -1 to day 6 and the change of [^18^F]FLT T/B ratio between day 0 and day 2 (linear correlation; Spearman Rank Order Correlation, r = 0.827, *P* = 0.0039).

**Figure 5:**
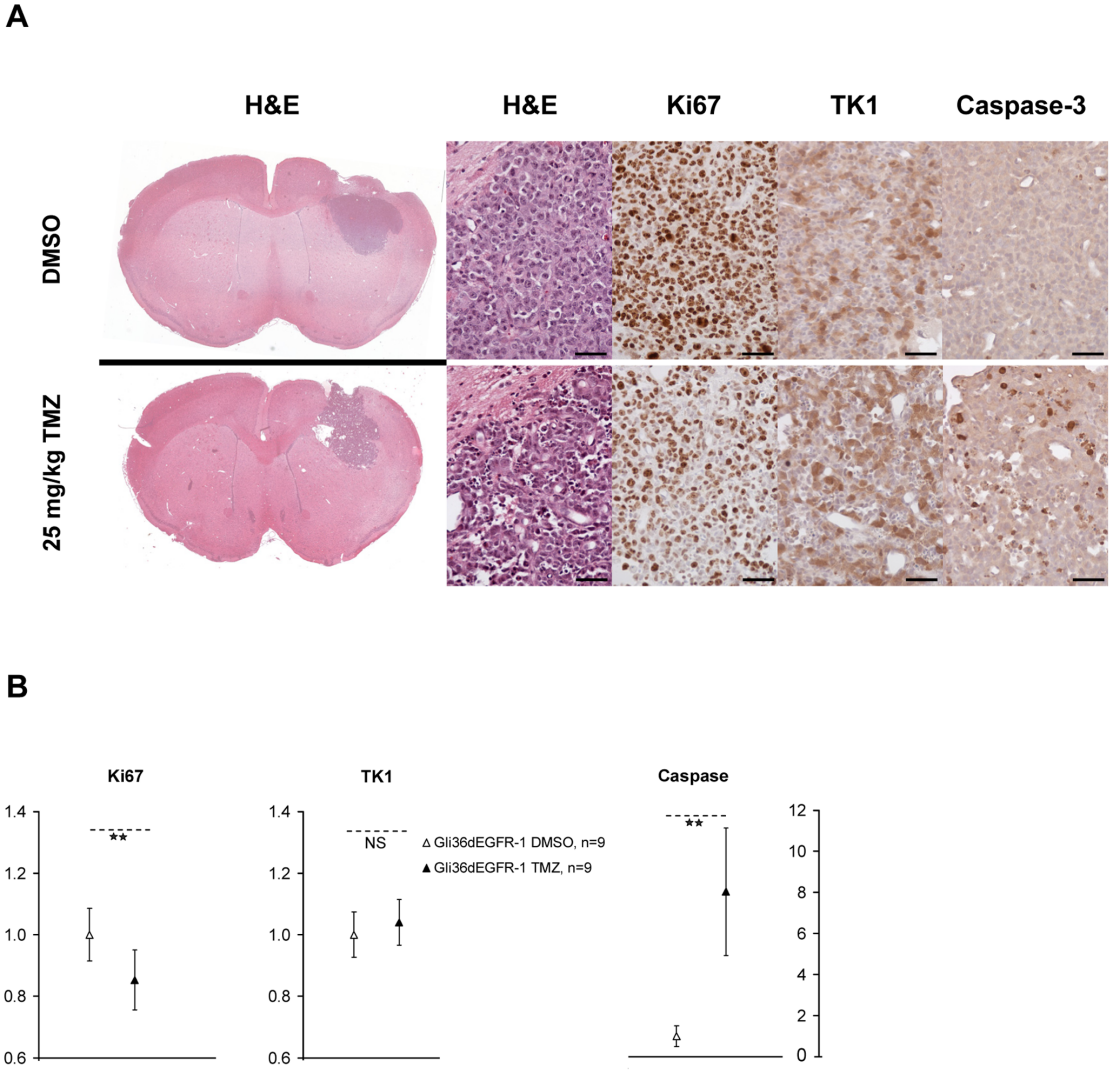
Immunohistochemistry of i.c. tumor tissue for H&E, Ki67, TK1 and active caspase-3 expression after 2 days of treatment. **A.** Tumor tissue sections were stained for H&E, Ki67, TK1 and active caspase-3 after 2 days of DMSO or 25 mg/kg TMZ treatment. Scale bars = 50 µm. **B.** Quantification of Ki67, TK1 and active caspase-3 staining. Differences between the treated (black triangles) and control (white triangles) groups were tested for significance using T-Test or Mann-Whitney Rank Sum Test (**: *P*<0.01, NS: not significant). Ki67 positive cells were less numerous in tumors treated with TMZ vs. DMSO. No significant difference could be observed on the TK1 staining. Active caspase-3 was induced in TMZ treated tumors.

Immunostaining for Ki67 at day 2 showed a significant reduction of the Ki67 positive cells in TMZ vs. DMSO treated tumors, in good agreement with the [^18^F]FLT-PET images (**Figure 5**). No significant change in TK1 staining could be observed. Active caspase-3 was up-regulated in TMZ vs. DMSO treated tumors.

## Discussion

In this study, we demonstrate that [^18^F]FLT-PET may serve as a noninvasive imaging parameter for an early readout of response of gliomas to TMZ chemotherapy. After 2 days of TMZ treatment, significant changes in [^18^F]FLT accumulation were observed in TMZ treated vs. control tumors, and a positive significant correlation between changes in [^18^F]FLT accumulation at early stage (day 2) and changes in tumor size later on (day 7) could be observed.

[^18^F]FLT-PET enables imaging and quantification of tumor cell proliferation *in vivo* [27]. Because the arrest of tumor proliferation is directly or indirectly induced by anti-tumor therapy, [^18^F]FLT-PET has raised considerable interest for follow-up after therapy. Different preclinical and clinical studies have shown the possibility to use [^18^F]FLT-PET as an early read-out of anti-tumor therapy before morphological changes become measurable in different types of tumors [27]. However, it soon became clear that [^18^F]FLT tumor uptake does not always correlate with the expression of proliferation markers such as Ki67 or TK1. [^18^F]FLT tumor accumulation is indeed influenced by intrinsic tumor thymidine levels which have been shown to differ in various tumor cell lines [28]. Furthermore, proliferating cells control the intracellular pool of thymidine required for DNA synthesis not only through the salvage pathway dependent on TK1, but also through the *de novo* synthesis pathway regulated by thymidylate synthase (TS). The balance between these two mechanisms may vary from one tumor type to another and could affect the relation between [^18^F]FLT tumor accumulation and tumor growth rate [29].

Therefore, whether [^18^F]FLT uptake as measured by PET could serve as an early read-out parameter of tumor response to therapy remains to be carefully evaluated with regards to the specific tumor type, but also with regards to the type of therapy itself. Depending on genetic alterations involved in cell cycle and growth regulation, DNA damage, for example, can lead to the halt of cell cycle progression either at the G1/S interface prior to TK1 cell cycle dependent upregulation, or at the G2/S interface allowing for transient accumulation of TK1 during S-phase [30]. In the present work, we used the Gli36dEGFR human glioma model in order to evaluate [^18^F]FLT-PET as predictive marker to TMZ therapy, the actual standard clinical chemotherapy for GBM [31]. A significant difference in terms of [^18^F]FLT T/B uptake variation was observed for the TMZ treated vs. the DMSO treated tumors as soon as on the second day of treatment. Furthermore, the variation of [^18^F]FLT T/B ratio at this very early time point was a good indicator for the variation of the tumor size at a later time point. The decrease of [^18^F]FLT uptake was particularly pronounced in the orthotopic GBM model, confirming the possible high potential of [^18^F]FLT-PET for the management of patients with brain tumors, due to the low [^18^F]FLT uptake in normal brain [12]. These results demonstrate that [^18^F]FLT-PET could be used as marker to assess a positive tumor response to TMZ therapy, and confirms a study on two patients affected by GBM and treated with TMZ [31]. Reduction of [^18^F]FLT uptake in the tumor has also been used as imaging biomarker to predict overall survival of patients with GBM treated with bevacizumab [32], irinotecan [33] and an mTor inhibitor [34].

However, 2 days after the start of treatment, the difference between Gli36dEGFR-1 and Gli36dEGFR-2 xenografts regarding [^18^F]FLT uptake was not significant. The human glioblastoma cell line used for this study, Gli36dEGFR, possesses a missense mutation in the *TP53* gene, rendering the protein inactive [23]. In p53-deficient tumor cells, DNA damaging agents can lead to transient increase of TK1 expression, as a result of G2 arrest due to checkpoint activation [30], which may limit the predictive value of [^18^F]FLT-PET regarding the very early scans. Therefore, for clinical applications, the time point when [^18^F]FLT-PET can be used to assess tumor response after treatment induction needs to be carefully evaluated. Finally, it should be mentioned that we performed static [^18^F]FLT imaging and analyzed the maximal [^18^F]FLT uptake. Improved imaging protocols, like dynamic acquisition and calculation of kinetic parameters, or improved quantification methods, like the measurment of the number of pixels above the 75th percentile, could further improve the predictive value of [^18^F]FLT-PET for TMZ efficacy.

In summary, even if the kinetics of the therapeutic response observed in this study cannot be directly translated into clinical application, our experimental data suggest that [^18^F]FLT-PET may have high potential to monitor early effects of TMZ therapy in patients with GBM.

## Supporting Information

Figure S1 **In vitro TMZ mediated cytotoxicity in human Gli36dEGFR-1 and Gli36dEGFR-2 glioma cells. A.** Pictures of surviving Gli36dEGFR-1 and Gli36dEGFR-2 colonies exposed to different concentration of TMZ (stained with crystal violet). **B.** Quantification of the clonogenic survival assay (significant difference between the two cell lines; **: *P*<0.001, Two-Way ANOVA). **C.** Whole-cell lysates were subjected to immuno-blotting with the MGMT and MSH6 antibodies. LN18 and Hela cell lysates served as positive control for MGMT and MSH6, respectively. MGMT was not observed in Gli36dEGFR-1 and Gli36dEGFR-2 cells. MSH6 was reduced in Gli36dEGFR-2 cells compared to Gli36dEGFR-1 cells, which may be a possible explanation for the observed lower TMZ sensitivity of the Gli36dEGFR-2 vs. the Gli36dEGFR-1 cells.
(TIF)

Figure S2 **Tumor size and [18F]FLT tumor uptake variation in s.c. xenografts after 7 days of TMZ treatment.** Tumor growth and variation of [^18^F]FLT T/B uptake ratio in Gli36dEGFR-1 xenografts (DMSO: n_tumor_ = 6 in n_mouse_ = 4; TMZ 25 mg/kg: n_tumor_ = 5 in n_mouse_ = 3; TMZ 50 mg/kg: n_tumor_ = 4 in n_mouse_ = 2) and in Gli36dEGFR-2 xenografts (DMSO: n_tumor_ = 5 in n_mouse_ = 4; TMZ 25 mg/kg: n_tumor_ = 7 in n_mouse_ = 4; TMZ 50 mg/kg: n_tumor_ = 4 in n_mouse_ = 2) were studied using [^18^F]FLT-PET/CT. **A.** Representative co-registered [^18^F]FLT-PET/CT coronal images of mice bearing Gli36dEGFR-1 (G−1) and Gli36dEGFR-2 (G−2) xenografts before (day 0) and after (day 7) daily injection of either DMSO or TMZ. **B.** Treatment with TMZ induced after 7 days a significant and dose dependant reduction of tumor size for the Gli36dEGFR-1 and the Gli36dEGFR-2 groups (Kruskal-Wallis One Way Analysis: *P* = 0.002, *P* = 0.006, respectively; Pairwise comparison: *: *P*<0.05, **: *P*<0.01). **C.** At day 7 a significant and dose dependant reduction of the [^18^F]FLT T/B uptake compared to day 0 was observed for the Gli36dEGFR-1 group, but not for the Gli36dEGFR-2 group despite reduction of the tumor size (Kruskal-Wallis One Way Analysis: *P* = 0.006, *P* = 0.114, respectively; Pairwise comparison: *: *P*<0.05, **: *P*<0.01). **D.** Immunohistochemistry of glioma tissue for Ki67 and TK1 expression. After 7 days of daily injection of DMSO, 25 mg/kg TMZ or 50 mg/kg TMZ mice were sacrificed and xenografts were fixed in PFA. Tissue sections were stained for Ki67 and TK1 expression. TMZ treatment induced a strong reduction of Ki67 and TK1 expressions in the Gli36dEGFR-1 group, whereas only a limited reduction could be observed for the Gli36dEGFR-2 xenografts. Scale bars = 50 µm.
(TIF)

Figure S3 **CT-determined size does not indicate GBM response to TMZ after 2 days of treatment in s.c. xenografts. A.** Quantitative analysis of the change of size ratios between days 0 and 2 in mice receiving daily injection of DMSO (Gli36dEGFR-1 n_tumor_ = 4 in n_mice_ = 4; Gli36dEGFR-2 n_tumor_ = 5 in n_mice_ = 5) or of 25 mg/kg TMZ (Gli36dEGFR-1 n_tumor_ = 5 in n_mice_ = 4; Gli36dEGFR-2 n_tumor_ = 3 in n_mice_ = 3). Differences between the treated group and the control group were not significant (T-Test or Mann-Whitney Rank Sum Test; NS: not significant). **B.** A positive correlation was observed between changes in CT-determined size between day 0 and day 2 and between day 0 and day 7 (Spearman correlation analysis; not significant).
(TIF)
