# Supplementary figures and images for: Early Assessment of the Efficacy of Temozolomide Chemotherapy in Experimental Glioblastoma Using [18F]FLT-PET Imaging
Source: PLoS One. 2013 Jul 4;8(7):e67911. doi: 10.1371/journal.pone.0067911 (PMC3701682; doi:10.1371/journal.pone.0067911)

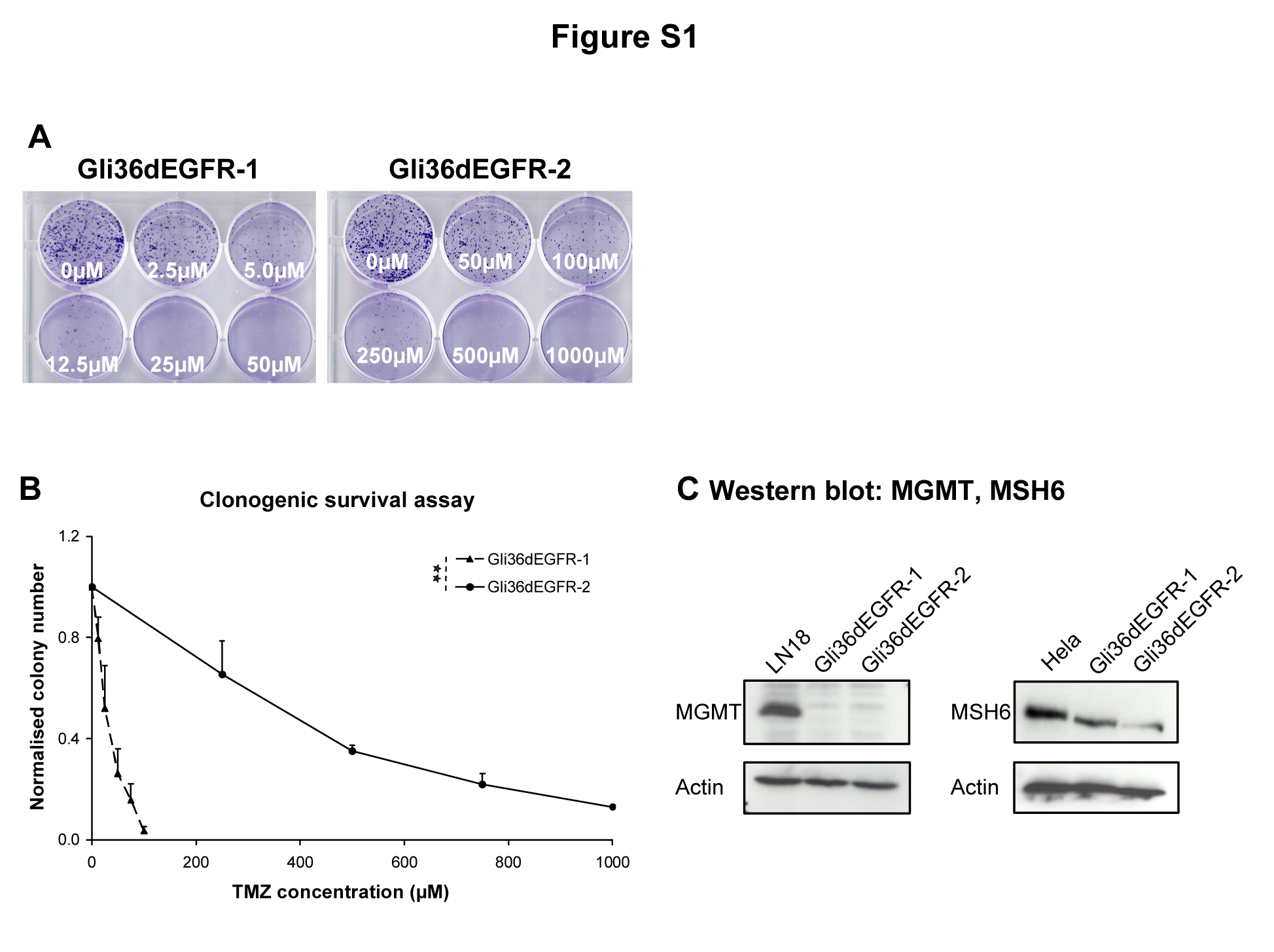

Supplement: Figure S1 — In vitro TMZ mediated cytotoxicity in human Gli36dEGFR-1 and Gli36dEGFR-2 glioma cells. A. Pictures of surviving Gli36dEGFR-1 and Gli36dEGFR-2 colonies exposed to different concentration of TMZ (stained with crystal violet). B. Quantification of the clonogenic survival assay (significant difference between the two cell lines; **: P<0.001, Two-Way ANOVA). C. Whole-cell lysates were subjected to immuno-blotting with the MGMT and MSH6 antibodies. LN18 and Hela cell lysates served as positive control for MGMT and MSH6, respectively. MGMT was not observed in Gli36dEGFR-1 and Gli36dEGFR-2 cells. MSH6 was reduced in Gli36dEGFR-2 cells compared to Gli36dEGFR-1 cells, which may be a possible explanation for the observed lower TMZ sensitivity of the Gli36dEGFR-2 vs. the Gli36dEGFR-1 cells. (TIF) [file pone.0067911.s001.tif]

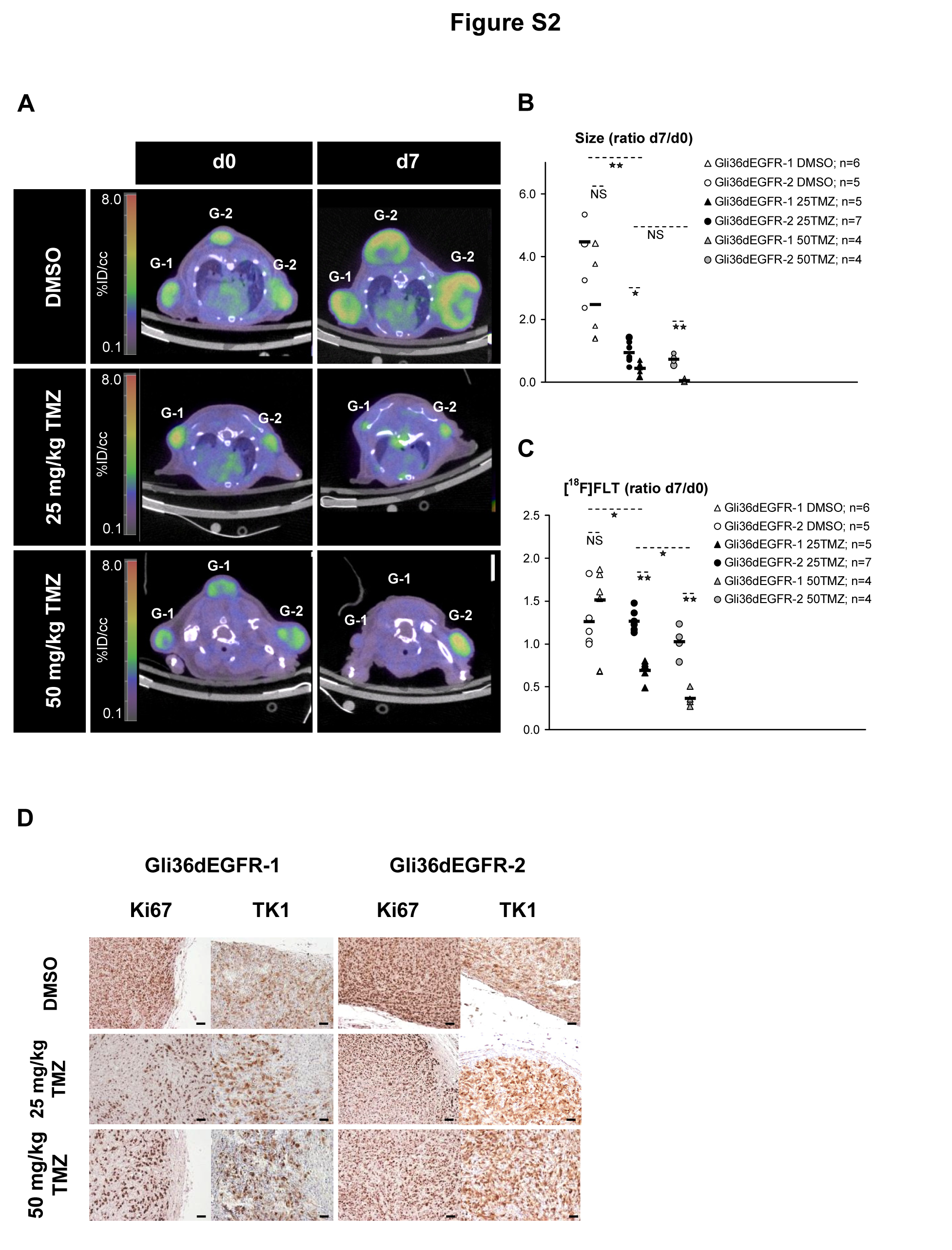

Supplement: Figure S2 — Tumor size and [18F]FLT tumor uptake variation in s.c. xenografts after 7 days of TMZ treatment. Tumor growth and variation of [18F]FLT T/B uptake ratio in Gli36dEGFR-1 xenografts (DMSO: ntumor = 6 in nmouse = 4; TMZ 25 mg/kg: ntumor = 5 in nmouse = 3; TMZ 50 mg/kg: ntumor = 4 in nmouse = 2) and in Gli36dEGFR-2 xenografts (DMSO: ntumor = 5 in nmouse = 4; TMZ 25 mg/kg: ntumor = 7 in nmouse = 4; TMZ 50 mg/kg: ntumor = 4 in nmouse = 2) were studied using [18F]FLT-PET/CT. A. Representative co-registered [18F]FLT-PET/CT coronal images of mice bearing Gli36dEGFR-1 (G−1) and Gli36dEGFR-2 (G−2) xenografts before (day 0) and after (day 7) daily injection of either DMSO or TMZ. B. Treatment with TMZ induced after 7 days a significant and dose dependant reduction of tumor size for the Gli36dEGFR-1 and the Gli36dEGFR-2 groups (Kruskal-Wallis One Way Analysis: P = 0.002, P = 0.006, respectively; Pairwise comparison: *: P<0.05, **: P<0.01). C. At day 7 a significant and dose dependant reduction of the [18F]FLT T/B uptake compared to day 0 was observed for the Gli36dEGFR-1 group, but not for the Gli36dEGFR-2 group despite reduction of the tumor size (Kruskal-Wallis One Way Analysis: P = 0.006, P = 0.114, respectively; Pairwise comparison: *: P<0.05, **: P<0.01). D. Immunohistochemistry of glioma tissue for Ki67 and TK1 expression. After 7 days of daily injection of DMSO, 25 mg/kg TMZ or 50 mg/kg TMZ mice were sacrificed and xenografts were fixed in PFA. Tissue sections were stained for Ki67 and TK1 expression. TMZ treatment induced a strong reduction of Ki67 and TK1 expressions in the Gli36dEGFR-1 group, whereas only a limited reduction could be observed for the Gli36dEGFR-2 xenografts. Scale bars = 50 µm. (TIF) [file pone.0067911.s002.tif]

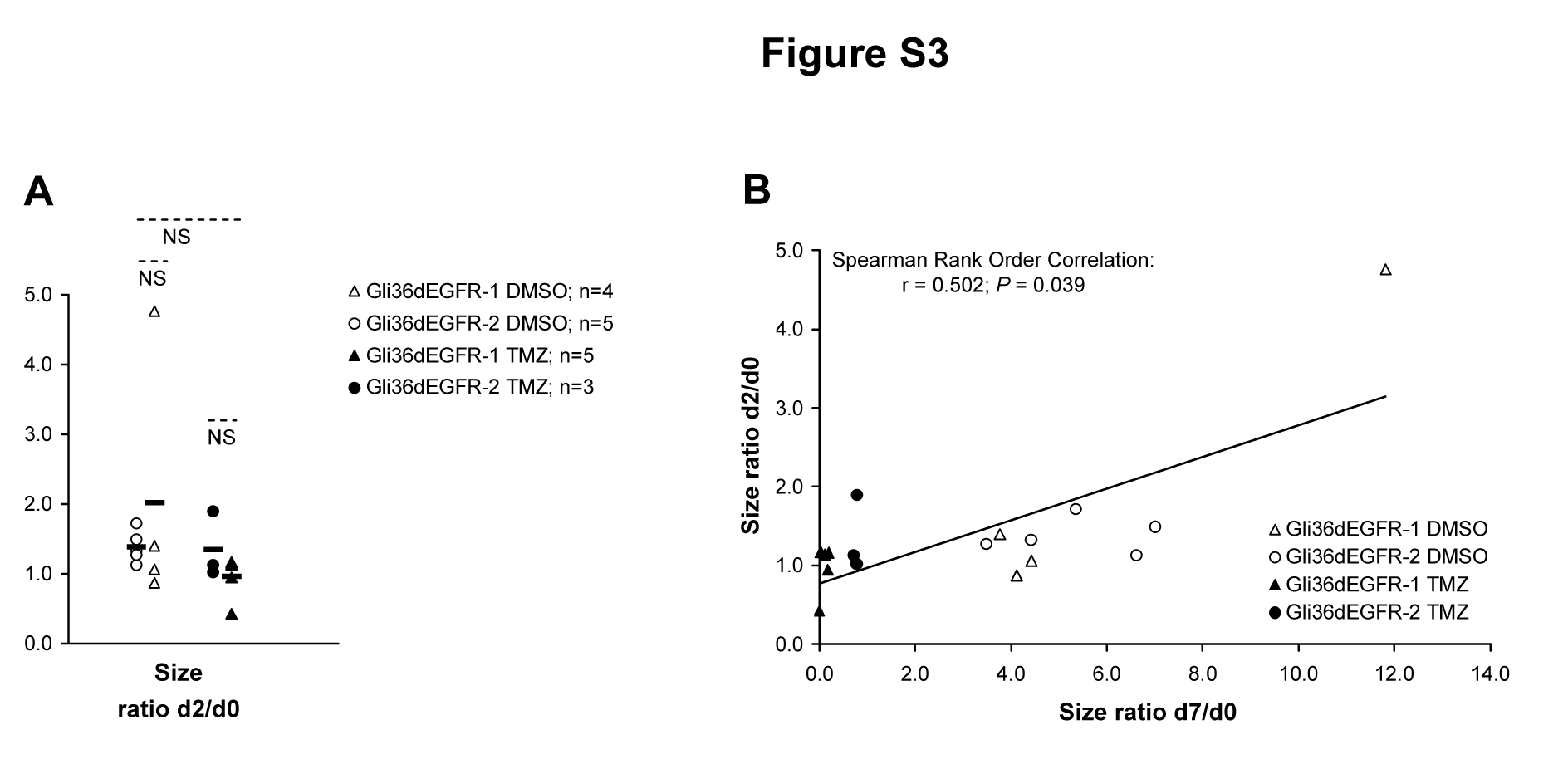

Supplement: Figure S3 — CT-determined size does not indicate GBM response to TMZ after 2 days of treatment in s.c. xenografts. A. Quantitative analysis of the change of size ratios between days 0 and 2 in mice receiving daily injection of DMSO (Gli36dEGFR-1 ntumor = 4 in nmice = 4; Gli36dEGFR-2 ntumor = 5 in nmice = 5) or of 25 mg/kg TMZ (Gli36dEGFR-1 ntumor = 5 in nmice = 4; Gli36dEGFR-2 ntumor = 3 in nmice = 3). Differences between the treated group and the control group were not significant (T-Test or Mann-Whitney Rank Sum Test; NS: not significant). B. A positive correlation was observed between changes in CT-determined size between day 0 and day 2 and between day 0 and day 7 (Spearman correlation analysis; not significant). (TIF) [file pone.0067911.s003.tif]
